# The role of nurse successional stages on species‐specific facilitation in drylands: Nurse traits and facilitation skills

**DOI:** 10.1002/ece3.3962

**Published:** 2018-04-27

**Authors:** Marina Fagundes, Wolfgang Weisser, Gislene Ganade

**Affiliations:** ^1^ Restoration Ecology Research Group Department of Ecology Universidade Federal do Rio Grande do Norte Natal RN Brazil; ^2^ Terrestrial Ecology Research Group Department of Ecology and Ecosystem Management School of Life Sciences Weihenstephan Technical University of Munich Freising Germany

**Keywords:** Caatinga, competition, degraded land, drought, pairwise interactions, positive interactions, successional stage, survival

## Abstract

Plant establishment is a challenge in semiarid environments due to intense and frequent drought periods. The presence of neighboring trees (nurses) can increase the establishment of seedlings (targets) by improving resource availability and microclimate. The nurse effect, however, might vary depending on nurse‐target species combinations but factors that predict this specificity are poorly known. We used a multispecies experiment to investigate the facilitation potential of trees from a range of successional stages, focusing on how nurse functional traits can predict species‐specific interaction outcomes. We conducted a factorial field experiment in a Brazilian semiarid tropical forest during a severe drought period. Sixty pairs of interacting tree species, 20 potential nurses, and three targets were used. Seedlings of all targets were planted both under and far from the nurse canopy, in a randomized block design replicated five times. Target growth and survival were monitored for 275 days from the beginning of the dry season, and interaction outcomes were calculated using the Relative Interaction Intensity (RII) index. Nurse functional traits such as successional stage, height, wood density, and canopy diameter were used as explanatory variables to predict RII values. The average effect of nurse species on target plants was in general positive, that is, seedling survival and growth increased under the nurse canopy. However, for growth pairwise interactions were significantly species specific. Successional stage was the only functional trait explaining RII values, with pioneer tree species being stronger facilitators than later successional trees. However, the explanation power of this variable was low, and positive, negative, or neutral interactions were found among nurse trees of all successional stages. Because seedling mortality during drought in semiarid systems is high, future studies should investigate how nurse traits related to water use could influence nurse facilitation skills.

## 1 INTRODUCTION

Facilitation is an important process that allows plant species to resist severe climatic conditions (Bagousse‐Pinguet et al., 2014; Cavieres et al., 2014). Facilitation occurs when one plant species, referred to as a “nurse,” improves the survival or growth of another “target” species, by expanding its realized niche (Soliveres et al., 2011), by ameliorating abiotic conditions (Jankju, 2013) or improving resource availability (Zou, Barnes, Archer, & McMurtry, 2005). Nurse species perform important roles in structuring plant communities at a global scale (McIntire & Fajardo, 2014; Soliveres & Maestre, 2014), and their effects are often reported in semiarid lands (Soliveres & Maestre, 2014). In dry lands, air temperatures and evapotranspiration from target species are lower underneath the nurse canopy (Jankju, 2013). Nurse plants can also alleviate water limitation for the whole plant community by performing hydraulic lift (Dawson, 1993; Pugnaire, Armas, Valladares, & Leps, 2003).

Nurse effects, however, might vary from positive to negative depending on the target species that establishes under nurse crown, and this process is referred as a species‐specific interaction outcome (Callaway, 1998; Callaway & Walker, 1997). Species‐specific interaction outcomes have been found to occur in a wide range of ecosystems (Landero & Valiente‐Banuet, 2010; Paterno, Siqueira, & Ganade, 2016; Poulos, Rayburn, & Schupp, 2014) and have been pointed out as a strong factor modulating seedling regeneration in plant communities (Paterno et al., 2016). However, predicting the outcome of nurse‐target interactions can be difficult, especially in high diversity ecosystems where multiple pairs of nurse and target species are able to interact. Nurse plants may also have positive effects on target survival but negative or neutral effects on growth (Gómez‐Aparicio, 2009; Paterno et al., 2016), making the interaction predictions even more complex. Therefore, there is an urgent need to identify the nurse traits that influence target performance. These factors have rarely been investigated because manipulative experiments connecting multiple species are scarce.

Some authors have pointed out that nurse‐target interaction outcomes could be predicted based on nurse species’ ecological strategies (Schöb, Armas, Guler, Prieto, & Pugnaire, 2013; Soliveres, Smit, & Maestre, 2015). For example, pioneer nurses in semiarid systems might have a higher tolerance to environmental stresses such as light intensity and drought (Kitao, Lei, Koike, Tobita, & Maruyama, 2000), which could affect conditions and resources provided to their neighbors (Diaz & Cabido, 2001). Pioneer nurses in an arid environment could deplete resources slower than late‐successional nurses by having stress‐tolerant features such as high wood density, and small size, which would allow them to establish in harsh or degraded areas (Grime, 1977). On the other hand, pioneer nurses could deplete resources faster than late‐successional nurses by exhibiting features related to high relative growth rate such as low wood density and large size, which would guarantee rapid colonization in open gaps (Kazakou, Vile, Shipley, Gallet, & Garnier, 2006). Therefore, nurses from different successional stages could have different effects on the same target species, a process that could partially explain species‐specific interaction outcomes.

The aims of this study were as follows: (1) To test the extent to which facilitation by nurse species occurs in a Brazilian semiarid system using a multispecies experiment. (2) To test whether nurse successional stage and morphological traits can predict facilitation and species‐specific interaction outcome. We expected facilitation to be frequent, although other interaction outcomes might occur. We also expected that nurse successional stage and morphological traits would explain facilitation skills because to establish in harsh semiarid areas, pioneer nurses might have evolved stress‐tolerant features that reduce their rates of resource uptake and consequently decrease their competitive ability (Grime, 1977).

## 2 MATERIALS AND METHODS

### 2.1 Study area

This study was conducted in the Caatinga semiarid tropical forest of Brazil. The vegetation is characterized by strong seasonality with an average precipitation around 700 mm/year, restricted to 4 months of rainy season, from February until June when rain is usually erratic. The mean temperature is 29°C, and soil temperature can reach 60°C during the dry season (Velloso, Sampaio, & Pareyn, 2002). Our study site was a degraded area once used for selective logging and cattle farming. Land use ended in 1950, after which forest recovery was allowed to take place. Forest structure comprises pioneer, early and late‐successional stage trees at the same site due to selective logging. The experimental site is now part of the “National Forest of Açu” protected area (Floresta Nacional de Açu, FLONA, ICMBio, RN) in Northeast Brazil (05°35′02,1″S–36°56′41,9″W).

### 2.2 Species interaction experiment

To test the effect of nurse on target species, we conducted a multi‐species experiment using 20 native nurse tree species and three native target tree species. A range of successional strategies was used to select nurse species. The strategies followed definition by Maia (2012) and varied from pioneer (trees that are the first to establish in open degraded areas), early‐successional (trees that establish in open degraded areas just after pioneer species have established), and late‐successional tree species (trees that rarely establish in open degraded areas). All tree species were commonly present at the site (Table 1).

**Table 1:** List of Caatinga tree species used in the nurse‐target interaction experiment and their successional stage based on Maia (2012). Mean ± 1 standard error of nurse traits: height, canopy diameter, and wood density were measured using three individuals of each nurse species

Family	Abbreviation	Nurse species	Successional stage	Height (m)	Canopy diameter (m)	Wood density
Bixaceae	C. vit	*Cochlospermum vitifolium*	Pioneer	6.66 **±** 0.33	4.7 ± 0.19	0.35 **±** 0.05
Burseraceae	C. lept	*Commiphora leptophloeos*	Pioneer	5.0 **±** 1.25	4.0 **±** 0.81	0.33 **±** 0.02
Combretaceae	C. lep	*Combretum leprosum*	Pioneer	3.50 **±** 0.86	3.2 **±** 0.40	0.75 **±** 0.02
Euphorbiaceae	C. bla	*Croton blanchetianus*	Pioneer	3.0 **±** 0.28	2.6 **±** 0.29	0.73 **±** 0.03
Fabaceae Mimosoideae	P. mon	*Pityrocarpa moniliformis*	Pioneer	6.83 **±** 1.40	5.26 **±** 1.26	0.76 **±** 0.03
Fabaceae—Papilionoideae	A. cea	*Amburana cearensis*	Pioneer	6.9 **±** 2.05	8.21 **±** 1.66	0.60 **±** 0.01
Fabaceae—Mimosoideae	M. ten	*Mimosa tenuiflora*	Pioneer	5.16 **±** 0.60	6.68 **±** 0.14	0.80 **±** 0.01
Fabaceae—Mimosoideae	P. sti	*Piptadenia stipulacea*	Pioneer	6.3 **±** 0.60	5.58 **±** 1.40	0.77 **±** 0.02
Apocynaceae	A. pyr	*Aspidosperma pyrifolium*	Early‐successional	6.83 **±** 0.92	6.20 **±** 1.06	0.69 **±** 0.04
Boraginaceae	C. glaz	*Cordia glazioviana*	Early‐successional	5.56 **±** 0.60	2.93 **±** 0.29	0.64 **±** 0.00
Capparaceae	C. has	*Cynophalla hastata*	Early‐successional	3.50 **±** .0.28	3.6 **±** 0.62	0.74 **±** 0.01
Erythroxylaceae	E. num	*Erythroxylum nummularia*	Early‐successional	5.16 **±** 0.88	1.82 **±** 0.73	0.84 **±** 0.00
Fabaceae—Caesalpinoideae	B. che	*Bauhinia cheilantha*	Early‐successional	4.16 **±** 0.66	3.65 **±** 0.92	0.79 **±** 0.00
Fabaceae—Caesalpinoideae	P. gar	*Poincianella gardneriana*	Early‐successional	4.33 **±** 1.16	5.88 **±** 0.94	0.87 **±** 0.02
Fabaceae—Mimosoideae	A. col	*Anadenanthera colubrina*	Early‐successional	5.80 **±** 2.10	6.00 **±** 3.00	0.80 **±** 0.05
Fabaceae—Caesalpinoideae	L. fer	*Libidibia ferrea*	Early‐successional	4.53 **±** 0.03	7.65 **±** 0.81	0.77 **±** 0.35
Malvaceae	P. mar	*Pseudobombax marginatum*	Late‐successional	6.00 **±** 0.86	3.96 **±** 0.85	0.29 **±** 0.01
Euphorbiaceae	S. mac	*Sebastiania macrocarpa*	Late‐successional	5.16 **±** 0.88	1.82 **±** 0.73	0.75 **±** 0.00
Bignoniaceae	H. imp	*Handroanthus impetiginosus*	Late‐successional	5.50 **±** 0.50	5.85 **±** 0.45	0.83 **±** 0.03
Anacardiaceae	S. tub	*Spondias tuberosa*	Late‐successional	7.66 **±** 0.72	16.11 **±** 1.19	0.57 **±** 0.03
**Target species**						
Fabaceae—Caesalpinoideae	P. pyr	*Poincianella pyramidalis*	Pioneer			
Fabaceae—Mimosoideae	A. col	*Anadenanthera colubrina*	Early successional			
Anacardiaceae	M. uru	*Myracrodruon urundeuva*	Late‐successional			

We chose nurse individuals spread in a radius of 1 km around FLONA de Açu's head office with distance between nurses varying from 2.5 to 1,200 m. Selection of nurse trees was based on the following criteria: (1) tree trunk larger than 10 cm circumference at breast height and; (2) isolated individuals to avoid neighbor interference. Nurse species height and canopy diameter were similar among species from different successional stages, but some variation within successional stages was allowed (Table 1). Three target species, *Poincianella pyramidalis* (Tul.) L.P Queiroz*, Anadenanthera colubrina* (Vell.) Brenan, and *Myracrodruon urundeuva* Allemão*,* were selected. Target selection was based on the following criteria: (1) all species were native and widespread in the Caatinga vegetation; (2) they embrace distinct successional stages; (3) they occurred naturally in the study area; and (4) they were available in commercial greenhouses in sufficient numbers to conduct the experiment. Target individuals were 6 months old and were, on average, 20 cm ± 0.5 tall at the start of the experiment. Species in Caatinga have evolved to have a high growth rate, because of the short rainy season. Thus, it is realistic for young plants to reach 20 cm height during the rainy season.

“Nurse” and “No nurse” treatments were arranged in a block consisting of one nurse plant individual and six target plants, with one individual target species in each treatment (Appendix 1). Blocks were replicated five times for each of the 20 nurse species, resulting in 100 nurse trees in total (100 blocks) and 600 target individuals. Once nurse tree individuals were chosen, a 2 m × 2 m plot was marked around each nurse tree. All vegetation present, commonly herbaceous species, was weeded before target planting. The same weeding treatment was performed in a 2 m × 2 m “no nurse” treatment plot that was located at a distance of 2.5 m from the nurse plot and was free from any other plant canopy influence. Target saplings were placed approximately 40 cm from the trunk of each nurse plant individual. We counted the number of leaves of target seedlings before planting. Immediately after planting, each target received 2 L of water. There was no further irrigation, but all targets were visited twice during the first week after planting and no plant died during this period.

### 2.3 Monitoring survival and growth

The experiment began on June 2014, toward the end of the rainy season. We used the dry season because during the rainy season, nurse plants effects can be masked by high water availability. Growth and survival of targets were recorded once a week in the first 2 weeks and then every 15 days for 85 days until August 2014, when all targets lost their leaves. Targets were then monitored once more in March 2015, 1 month after the start of the following rainy season.

We recorded growth by counting the number of leaves flushed at each inspection. The number of leaves was used instead of height, because height can remain unchanged in this semiarid system during early growth, when seedlings allocate most of their biomass to roots. Leaf flushing, on the other hand, is strongly responsive to environmental stress. Seedlings under stressful conditions would lose their leaves and avoid further flushing, but they can quickly flush new leaves once environmental conditions are improved (Lima & Rodal, 2010). Due to the high number of target individuals in the experiment, we did not mark leaves to follow their individual fate. For each survey, we used number of leaves produced in relation to the initial number of leaves registered at the beginning of the experiment. We thus calculated the percentage of leaves gained or lost relative to the number of leaves that the target had when planted. Therefore, the measure of leaves gained in each survey was used as a proxy of growth through time, whereby leaf loss indicates a stress response, whereas leaf flushing indicates lack of stress. Because species replace their leaves regularly, values <100 do not represent lack of leaves, but, rather, that the rate of leaf renewal was smaller than the rate of leaf loss.

We also checked for survival of target plants at each inspection. When an individual lost all its leaves, we tested for mortality by scratching the bark carefully to check whether its tissue was still green or fresh. The survival response variable represented the number of days a given target was able to survive after being planted. The maximum survival days were set by the total duration of the experiment, 275 days.

### 2.4 Nurse trait measurements

We collected nurse traits from three individuals of each nurse species. For each individual, we estimated height and canopy diameter and measured wood density. Canopy diameter represented the average length of two perpendicular axes that were placed on the tree crown facing north and south. We measured wood density by sampling one branch from each tree, removing its bark and applying the water displacement method performed by Pérez‐Harguindeguy et al. (2013).

To calculate the effect of a nurse species on a target species growth and survival, we calculated the pairwise Relative Interaction Intensity—RII (Armas, Ordiales, & Pugnaire, 2004):

$$\text{RII}=\frac{B_{w}-B_{o}}{B_{w}+B_{o}},$$

where *B*_w_ represents target performance under the nurse, and *B*_o_ represents target performance in the “no nurse” plot. The RII values range from −1 to +1; whereby negative values indicate competitive interactions (negative effect of nurse on target) and positive values indicate facilitation (positive effect of nurse on target). For survival, we calculated one RII‐value for each of the three target species, in each block. For growth, the same calculations were performed separately for each measurement recorded through time.

### 2.5 Statistical analysis

All analyses were performed in R (http://www.r-project.org, R Core Team, 2015, version 3.2.0) following the Zuur, Ieno, Walker, Saveliev, and Smith (2009) protocol. To understand the facilitation effect of different nurse tree species on target plants, we applied two generalized linear mixed models (GLMM) one using survival and the other using growth as the response variable. The GLMM used the “lmer” function in the “lme4” package (Bates et al., 2014), and the explanatory variables were nurse species, target species, and their interaction. Significance was established by log‐likelihood ratio tests removing each variable from the full model to calculate its overall effect. We used a normal error distribution for all analysis (Crawley, 2007).

To test whether nurse attributes can predict nurse facilitation effects, we performed a Linear Model Selection, following Crawley (2007). The variables growth and survival were used as response variables and nurse successional stage, height, canopy diameter, and wood density as explanatory variables. For growth, repeated measurements over time were included as a random factor nested within blocks to correct for temporal pseudo‐replication. For survival, there were no repeated measurements over time, and only block was considered a random factor.

## 3 RESULTS

### 3.1 Facilitation effect of nurses on targets

**Figure 1:**
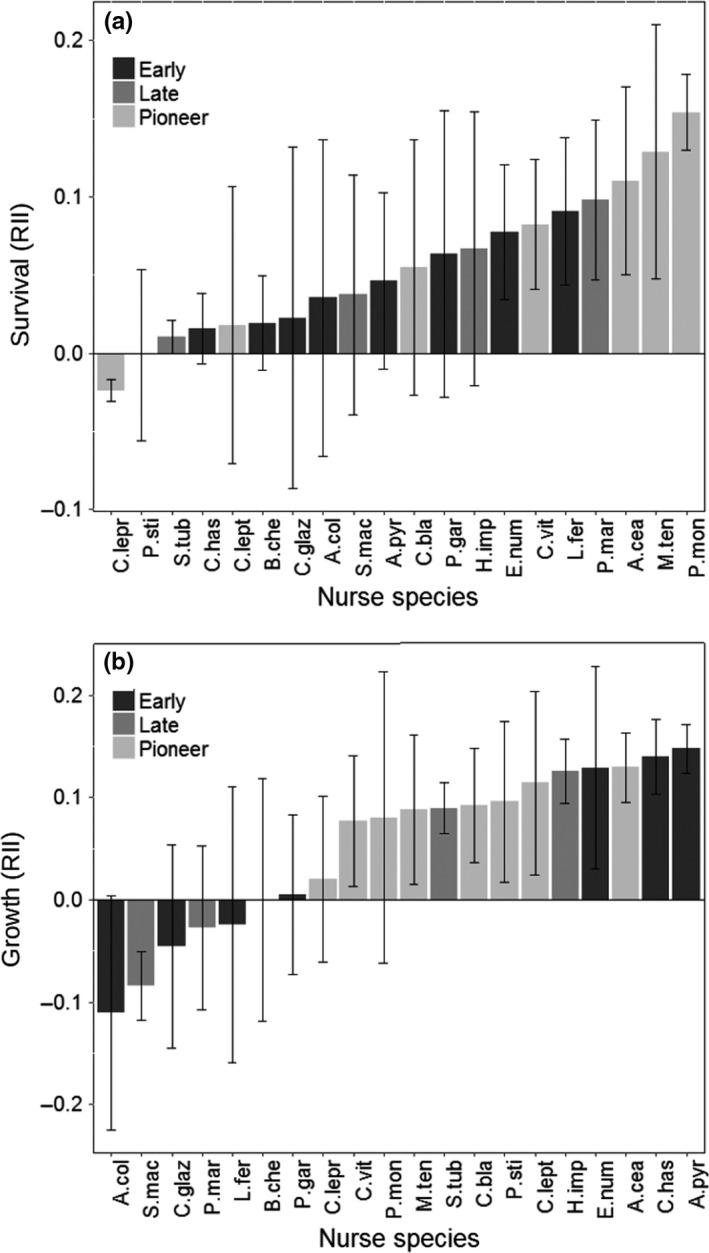
Average effects of 20 nurse species on three target species growth (a) and survival (b), measured using the RII index. Negative values indicate competitive interactions (negative effect of nurse on target, i.e., growth or survival is lower under the nurse canopy than outside the nurse canopy) and positive values indicate facilitation (positive effect of nurse on target, i.e., growth or survival is higher under the nurse canopy than outside the nurse canopy). Each bar represents the average effect of one nurse species across three target species replicated 15 times, error bars represent 1 standard error. The complete name of all species can be found on Table 1. RII, Relative Interaction Intensity

As expected, facilitation was common in the Caatinga semiarid tropical forest. For survival, 18 nurses showed positive average effects, that is, facilitation, and two nurses showed negative average effects, that is, competition (Figure 1a). The average increase in survival across all nurse plants was 8 days for the target *A. colubrina*, 7 days for the target *M*. *urundeuva* and 18 days for the target *P*. *pyramidallis*, that is, 2.9%, 2.6%, and 6.5%, respectively (Appendix 2). When only positive nurse‐target interactions were considered (i.e., where the presence of a nurse increased average target survival across the five replicates), the average increase in survival was 35 days (12%) for *A. colubrina*, 20 days (7.2%) for *M. urundeuva,* and 36 days (13%) for *P*. *pyramidallis*. It is important to highlight that despite the positive effect of nurses on targets, few target plants survived throughout the dry season. After 275 days, only 18 target individuals survived under nurse canopies and eight without a nurse.

For growth, the average nurse effect (average RII across all target species and replicates) was positive in 14 of 20 nurse species. One nurse showed, on average, a neutral effect (RII = 0), and five nurses had, on average, a negative effect on target growth (Figure 1b). All target species were able to flush new leaves in both “nurse” and “no nurse” treatments (Appendix 3).

### 3.2 Species‐specific relationship

**Figure 2:**
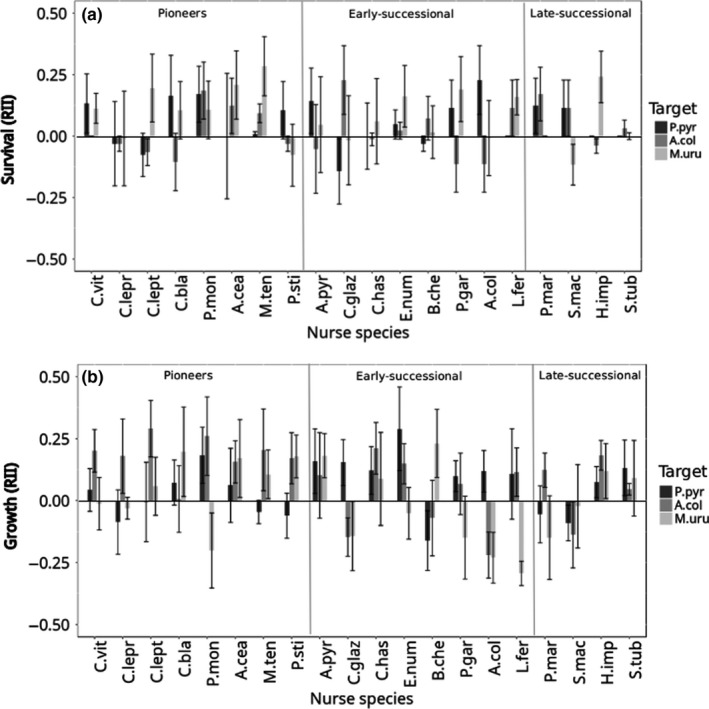
Effect of 20 nurse species on growth of the three different target species calculated using the Relative Interaction Index (RII). Nurses of all successional stages can affect positively or negatively target plants. Growth is measured as percentage of leaves produced during the experiment. Bars represent the average nurse effect on performance of each target, to Survival (a) and Growth (b) varying from −1 (competition) to 1 (facilitation) for each nurse‐target combination. Error bars represent ±1 standard error. The complete name of all species can be found on Table 1

For survival, we found no species specificity (Table 2). Despite the fact that few nurses exerted a consistent negative or positive effect on targets, there was no significant interaction between nurses and target species (χ^2^ = 44.804, *df* = 38, *p* = .207, Figure 2a). Only three of the 20 nurses, namely *Pityrocarpa moniliformis, Erythroxylum nummularia,* and *Mimosa tenuiflora*, facilitated all targets, and no nurse had negative effects (competition) on all targets.

**Table 2:** Table of linear mixed‐effect models of nurse effect on target survival and growth. The experiment consists of 20 Caatinga nurse trees and three target plant species replicated five times. Relative Interaction Intensity index—RII (Armas et al., 2004) used as response variables was calculated based on target survival (number of survival days) and target growth (proportion of leaves gained through time). The explanatory variables (fixed factors) are nurse species, target species, and their interactions. For growth measurements, time was nested in plot as a random factor

	Log‐likelihood	χ^2^	*df*	*p* Value
Survival				
Complete model	−16.9195			
Nurse × target	−5.4827	44.804	38	.2079
Nurse effect	−10.185	54.21	57	.5804
Target effect	−6.6636	47.166	40	.2029
Growth				
Complete model	−1,574.5			
Nurse × target	−1,647.0	144.93	38	<.001
Nurse effect	−1,672.9	196.84	57	<.001
Target effect	−1,654.2	159.28	40	<.001

RII, Relative Interaction Intensity.

With respect to growth, nurse‐target interactions were strongly species specific (Table 2, χ^2^ = 144.93, *df* = 38, *p* = <.001). Few nurse species exerted a consistent positive or negative effect on all target species (Figure 2b). From the 20 nurse species, only *Handroanthus impetiginosus* exerted positive effects on all target species, and only *Sebastiania macrocarpa* exerted negative effects on all targets species. All other nurse species exerted both positive and negative effects on target species. Moreover, target species also showed different responses when interacting with different nurse species (Figure 2b).

Net nurse effect also varied between target growth and survival. Positive effects on survival but negative effects on growth were, for example, found for the nurse *S. macrocarpa* when paired with target *A. colubrina*, and the combinations *P. moniliformis*–*M. urundeuva*, and *Pseudobombax marginatum*–*P. pyramidalis*. Positive effects on growth but negative effects on survival were found for the combinations *Poincianella gardneriana*–*A. colubrina* and *Piptadenia stipulacea*–*M. urundeuva* (Figure 2).

### 3.3 Effects of nurse traits on facilitation skills

**Figure 3:**
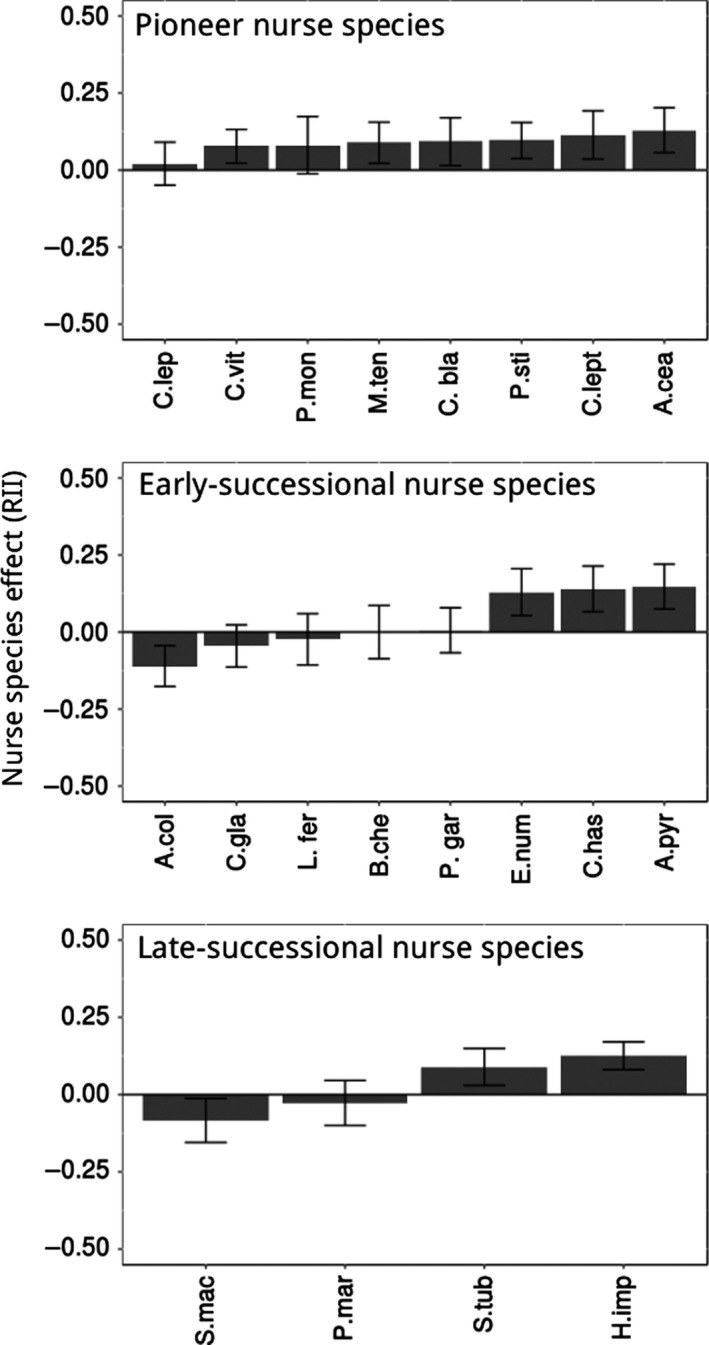
Average effect of nurse species on target growth calculated using RII for each successional stage. All successional stages present species‐specific interactions and potential to facilitation. Pioneer nurses present in average higher facilitative effects. Error bars represent ±1 standard error. The complete name of all species can be found on found on Table 1. RII, Relative Interaction Intensity

Nurse successional stage significantly explained RII values for target growth (*F* = 3.53, *df *= 2; 2,382, *p*‐value = .029, *r* ^2^ = .09), while the other variables height, canopy diameter, and wood density did not improve the model. Facilitation of target growth was more frequent and intense in average for nurses from pioneer successional stages than for other successional stages (Figure 3). Differences in effect size were, nevertheless, relatively small, and there was considerable variation in RII within a single successional stage (Figure 3). For the target survival model, neither nurse successional stage (*F* = 0.162, *df *= 2; 263, *p*‐value = .928), nurse height (*F* = 0.892, *df *= 1;263, *p*‐value = .269), canopy diameter (*F* = 0.362, *df *= 1; 263, *p*‐value = .547), and wood density had (*F* = 0.0002, *df *= 1; 263, *p*‐value = .964) a significant effect on RII values. Additionally, there was no match between nurse successional stage and target successional stage explaining RII values, so nurse species were able to both facilitate and compete with targets from all successional stages (Figure 2).

## 4 DISCUSSION

An important novelty of this work is that nurse tree successional stages can partially explain facilitation skills in this tropical dry forest, where nurse pioneer trees presented a strongest positive effect on targets than late‐successional species. Nurse successional stage, therefore, could partially explain species‐specific interactions. Our results also corroborate three process that are frequently reported in the literature: (1) Facilitation is a widespread process in harsh environments (Flores & Jurado, 2003; He, Bertness, & Altieri, 2013; Soliveres & Maestre, 2014); (2) Species‐specific interaction outcomes are common for semiarid biomes (Landero & Valiente‐Banuet, 2010; Paterno et al., 2016; Wright, Schnitzer, & Reich, 2014); and (3) Nurse positive effects are stronger on survival than growth, a general pattern found in different ecosystem types (Bertoncello, Oliveira, Hool, & Martini, 2016; Ganade & Brown, 2002; Gómez‐Aparicio, 2009).

### 4.1 Pioneers nurse effect

The reason why successional stages could partially explain tree facilitation skills might be related to the fact that pioneer species have evolved stress‐tolerance characteristics to establish in harsh arid ecosystems (Grime, 1977). In this case, pioneer species would deplete resources slower, making soil moisture available for longer periods, which would benefit target species establishing under their crowns. However, there was no evidence that nurse species had specific characteristics of stress‐tolerant species such as high wood density, low height, and small crown size. Additionally, these traits did not differ between successional stages nor did they influence facilitation. Even a key nurse trait such as crown size, which creates the microclimate amelioration for target species was of little importance for predicting facilitation (Soliveres, 2014; Zhang & Zhao, 2014). Although common morpho‐functional plant traits can be used to indicate competitive and stress‐tolerance strategies in semiarid lands (Graff & Aguiar, 2017), they might not be enough to elucidate the full complexity of nurse facilitation mechanisms in dry forests. Future studies should contemplate how physiological and morphological traits strongly related to water use such as rooting architecture or specific leaf area could influence nurse facilitation, species‐specific interactions.

This work shows that nurse successional stage could play a role in complex species‐specific interaction outcomes, which are frequently unpredictable (Anthelme, Meneses, Valero, Pozo, & Dangles, 2017). This might be due to differences in the way nurses from distinct successional stages alter conditions and available resources for the same target species (Diaz & Cabido, 2001). Interaction outcomes might also depend on how nurse strategies combine with different target needs (Holmgren, Gomez‐Aparicio, Quero, & Vallarades, 2012; Paterno et al., 2016; Woods & Miriti, 2016). For example, targets that are more prone to water stress are more likely to be facilitated by nurses that maintain water in the system, for example, by performing hydraulic lift or presenting high water use efficiency. In our work, there was no predictable match between nurse and target successional stages. Additionally, the explanatory power of the model was not strong, and alterations to interaction outcomes were found for all nurse successional stages. Therefore, the role of nurse successional stage on nurse performance should be considered with caution. Target and nurse morpho‐physiological traits that together predict the outcome of a particular interaction match should be investigated in the future to refine interaction outcome predictions.

### 4.2 Tropical dry forest dynamics

Our results emphasize that the Brazilian Caatinga is a harsh environment where drought is a strong force shaping plant recruitment. Despite generally positive nurse effects on survival (Bertoncello et al., 2016; He et al., 2013), drought was still the strongest force limiting regeneration (Jankju, 2013). It is important to understand that wet seasons can be very erratic in Caatinga, and seedlings have to reach a certain root size, and a minimum amount of storage to be able to keep themselves alive through dry periods until the next rain arrives. Therefore, any process that promotes higher probability of survival and growth should influence the seedlings’ chance to persist until water becomes available. Our results reinforce the importance of nurse species in a biome intensely limited by water supply in which the unpredictability of rain strongly influences seedling recruitment (Holmgren & Scheffer, 2001). Future understanding of the mechanisms that define a good nurse in tropical semiarid lands might reveal key factors to combat land degradation and desertification and improve programs of restoration and land management.

## CONFLICT OF INTEREST

None declared.

## AUTHOR CONTRIBUTIONS

Author FM, GG, and WW conceived the ideas led the writing of the manuscript; FM and GG designed methodology, collected the data, and analyzed the data; GG funded field work; WW funded international exchange trips. All authors contributed critically to the drafts and gave final approval for publication.

## DATA ACCESSIBILITY

Species‐specific interactions values: available through Dryad (http://datadryad.org/) after acceptance of the manuscript.

## APPENDIX 1

### Scheme of nurse‐target interaction experiment

Illustration of nurse‐target interaction experiment. Each block consists of two plots located around and far from a nurse plant. Nurse treatment plot (a), consisting of one sapling of each target species planted below the nurse canopy. Control treatment plot (b) consisting of one sapling of each target species planted 2.5 m far from any canopy influence. The experiment consisted of 20 nurse tree species replicated five times, and three target tree species, with a total of 100 blocks.

## APPENDIX 2

### Average number of survival days for each targets species under the nurse and far from nurse

Mean number of survival days for each target species under nurse (light bars) and no nurse (dark bars) treatments. Error bars represent the standard deviation. Each nurse‐target interaction was replicated five times.

## APPENDIX 3

### Percentage of leaves gained/lost during the time of experiment of all targets under and far from nurse

Percentage of leaves gained/lost during the time of experiment of all targets under and far from nurse. Targets growth measured as a percentage of leaves gained/lost through time. *A. columbrina* (pink), *M. urundeva* (green), and *P. pyramidalis* (blue). Solid lines represent target performance under the nurse, and dashed lines represent target performance far from nurse.
